# Changes in long-term care insurance revenue among service providers during the COVID-19 pandemic

**DOI:** 10.1186/s12913-024-10832-4

**Published:** 2024-04-13

**Authors:** Tomoko Ito, Xueying Jin, Makiko Tomita, Shu Kobayashi, Nanako Tamiya

**Affiliations:** 1 Institute of Medicine, University of Tsukuba, 1-1-1 Tennodai, Tsukuba, Ibaraki 305-8575 Japan; 2 Health Services Research and Development Center, University of Tsukuba, 1-1-1 Tennodai, Tsukuba, Ibaraki 305-8575 Japan; 3 Department of Social Science, National Center for Geriatrics and Gerontology, 7- 430 Morioka-cho, Obu, Aichi 474-8511 Japan; 4 Analysis & Innovation Dept., SMS Co., Ltd., Sumitomo Fudosan Shibakoen Tower, 2-11-1, Shibakoen, Minato-ku, Tokyo, 105-0011 Japan

**Keywords:** Long-term care services, Revenue, COVID-19

## Abstract

**Background:**

The COVID-19 pandemic has impacted peoples’ health-related behaviors, especially those of older adults, who have restricted their activities in order to avoid contact with others. Moreover, the pandemic has caused concerns in long-term care insurance (LTCI) providers regarding management and financial issues. This study aimed to examine the changes in revenues among LTCI service providers in Japan during the pandemic and analyze its impact on different types of services.

**Methods:**

In this study, we used anonymized data from “Kaipoke,” a management support platform for older adult care operators provided by SMS Co., Ltd. Kaipoke provides management support services to more than 27,400 care service offices nationwide and has been introduced in many home-care support offices. The data used in this study were extracted from care plans created by care managers on the Kaipoke platform. To examine the impact of the pandemic, an interrupted time-series analysis was conducted in which the date of the beginning of the pandemic was set as the prior independent variable.

**Results:**

The participating providers were care management providers (*n* = 5,767), home-visit care providers (*n* = 3,506), home-visit nursing providers (*n* = 971), and adult day care providers (*n* = 4,650). The results revealed that LTCI revenues decreased significantly for care management providers, home-visit nursing providers, and adult day care providers after the COVID-19 pandemic began. The largest decrease was an average base of USD − 1668.8 in adult day care.

**Conclusion:**

The decrease in revenue among adult day care providers was particularly concerning in terms of the sustainability of their business. This decrease in revenue may have made it difficult to retain personnel, and staff may have needed to be laid off as a result. Although this study has limitations, it may provide useful suggestions for countermeasures in such scenarios, in addition to support conducted measures.

## Background

The COVID-19 pandemic has changed many peoples’ health-related behaviors. In particular older adults, who were the most serious victims of COVID-19 [1, 2], had their activities restricted in order to avoid contact with people [3, 4]. A previous quasi-experimental study revealed that the number of users of long-term care insurance (LTCI) services was influenced by the pandemic [5]. A decrease in the number of users of adult day care was particularly evident. At the same time, the decrease in service utilization has simultaneously affected the provider management practices of the service providers [6]. 

The LTCI system was launched in Japan in 2000. In 2000, the LTCI system was established to follow the rapid aging. The LTCI system ensures two main types of services: services for convalescent older adults who live at home and services for those who stay in facilities. For those who live at home, there are also two types of services: home-visit services, in which professionals go to the home of the patient to provide services, and day-care services, in which the patient himself/herself goes to the facility during the daytime to receive services at the facility. Most users of LTCI services pay 10% of the total cost of these services out-of-pocket, and the rest is covered by pooled LTCI premiums and taxes. In part, users with sufficient income are asked to pay up to 30% of the total cost.

Under this system, private providers were allowed to provide long-term care services, covered by public insurance [7, 8]. This change opened up a market that had previously only been available to providers in a public capacity [9]. As a result, the number of providers of LTCI services has been increasing every year. For example, the number of facilities providing adult day cares increased from 8,037 in 2000, when the system was first established, to 24,087 in 2020 [10]. The increase in the number of such providers has also led to an increase in accessibility of use, helping to better maintain the daily lives and activities of those who need care. On the other hand, in the LTCI business, revenue strongly depends on the public’s use of the services. When a user quits using LTCI services, it has a significant impact on the revenue of the provider. In such cases, service management can easily deteriorate, and providers may sometimes close immediately after they have been established.

During the pandemic, some offices were forced to close due to infection control, and there were concerns about managerial difficulties in terms of maintaining the revenues by providing publicly insured LTCI services in Japan. With declining revenues, which lead to unstable finances for establishments and increased risks of bankruptcy [11], there have been a number of Japanese studies showing the impact [12] of the pandemic on revenues. That report [12] revealed about 40% of providers faced a reduction in revenue and also they predicted that reduction continuously. However, there were few studies to show the decline in revenues of LTCI providers with the nationwide scope in Japan. There have also been investigations regarding service scope, in terms of differences in the variety of services offered due to the pandemic’s impact. In a previous study, the decline in number of users was different from that of LTCI services. This present study could suggest the future decline in revenue with the other pandemics or some disasters, and the necessity to consider the preventive measurement for the stopping of services provided. For that future consideration, firstly, the descriptive study to reveal the impact of the pandemic on the LTCI revenue was needed for the current situation with few previous studies. Additionally, time series analysis was effective to capture the impact on the change of revenue compared with the former trend. Therefore, this study aimed to verify the change in revenue experienced by LTCI among service providers in Japan during the COVID-19 pandemic, for a variety of services.

## Methods

### Study subjects

In this study, we used anonymized data from “Kaipoke,” a management support platform for older adult care operators provided by SMS Co., Ltd. Kaipoke provides management support services to more than 27,400 care service offices nationwide and has been introduced in many home-care support offices. Study subjects were the LTCI service providers including care management, home-visit care, home-visit nursing, and adult day care which had provided their services in November 2018. We followed up on the subjects’ monthly revenue from December 2018 to November 2020. Kaipoke supports the office works of LTCI service providers including the requirement of revenue, therefore, we could extract the data.

Each service provider of the LTCI system in Japan was certified by a municipal body established by the providers. The municipal body examined the standards for personnel, facilities, and equipment of providers in accordance with the standards set by the national government. Certified providers can provide services for disabled older adults, and claim the expenses to the city as a public insurer.

### Measures

#### Outcome

The outcome of this study was the revenue of LTCI services claimed by the provider. Revenue was recorded in Japanese yen. The exchange rate was set at an average of 113 USD in November 2018, to show global results. The revenue from LTCI services was discussed and revised by the subcommittee of the Ministry of Health, Labor, and Welfare once every three years. In recent years, against the backdrop of a shortage of LTC personnel, restructuring within LTCI companies has been increasing. Revenue was standardized throughout the country in the form of “units.” The units were determined on a regional basis between 0.09 USD and 0.10 USD, reflecting the differences in prices and other factors in the region. The LTCI service providers in the region received revenues in yen based on the conversion of the determined unit [13]. For example, the unit for one service of between 30 min and one hour in home-visit care, mainly with physical assistance, was 394 units in 2018. In the 23 wards of Tokyo, the area with the highest billing rate, this would have been billed at 0.10 USD per unit, for a total of 39.7 USD. The lowest billing rate is 0.09 USD per unit, which would have come out to 34.9 USD for the same service, a difference of 4.8 USD. We used the amount reflecting this regional difference as the outcome.

In this study, the outcome was the amount of revenue received by providers for four types of services: care management, home-visit care, home-visit nursing, and adult day care. The revenue was based on the revenues made in the 2018 revision. Care management [14] was a service done by a “care manager,” who would make the care plan and prepare the LTC schedule, in liaison and coordination with service providers. A care manager was the only official person to make the care plan, except in the cases of families with qualified members who had completed a certain level of experience and training in care management, and had successfully completed an examination [10, 15]. The most recent results showed that care management was paid at 125.7 USD per month per user [16]. Home-visit care was a service in which a trained person visited the user’s home to provide daily living care such as cleaning and laundry, and physical care such as bathing and toileting. This service is the most widely used home-visit service, with approximately one million users nationwide. Per capita usage was 673.5 USD per month in 2018. Home-visit nursing was primarily provided by nurses. This service included the management of chronic disease conditions or the maintenance of medical treatments such as catheterization. The amount of revenue per user for home-visit nursing was 426.5 USD per month on average. Finally, adult day care was a service that assisted in daily care and training to maintain or improve the user’s physical or psychological functions at the facility, rather than at home. Users took shared buses or private cars to the facility, where several users gathered for activities, meals, and bathing. This care was provided to relieve the users’ senses of social isolation, maintain the users’ physical and psychological functions, and reduce the physical and psychological burdens on the users’ families. The number of adult day care users was the largest among the community-based services, at 1.13 million. The amount of monthly revenue for the usage of this service in 2018 was 820.4 USD.

Certified providers were billed for LTCI on a monthly basis. In this study, the outcome was the amount claimed by each provider for each type of service in the 24 months from December 2018 to November 2020.

#### Exposures

The exposure in this study was the new coronavirus infection pandemic. In Japan, the first case of infection was recorded on January 16, 2020. The number of infected individuals has gradually increased since then. The first wave in Japan was confirmed in April 2020, and the daily number of positive cases in the first wave was 720. At that time, little was known about the new coronavirus. Japan was in a state of panic and required people to stay at home. This was followed by a second wave in August 2020 with more than 1,500 positive cases per day. Therefore, in this study, we considered the exposure period as beginning in January 2020, when the pandemic started in Japan.

### Statistical analysis

The distribution of monthly revenues of LTCI service providers was described in a time series between December 2018 and November 2020. A summary of the distribution is shown as the means and medians of the revenues (unit: USD). To assess this trend, an interrupted time-series analysis was conducted [17], in which the outcome was the trend in the amount of monthly revenues of LTCI services (Yt). This analysis was established to observe the interruption of the outcome variable level and trend through the equally spaced periods before and after the introduction of an intervention. In the present study, the intervention of analytical framework was defined for the pandemic of COVID-19. Therefore, we tried to extract the interruption by the pandemic on the LTCI revenue trend. In addition, for that analysis, the assumption was needed that the influences on the outcome variable were stable through the observed periods. We also followed that assumption. The periods were divided into pre-COVID-19 (December 2018-December 2019) and post-COVID-19 (January 2020-November 2020) periods. During the pandemic, the Japanese government issued a declaration of emergency across seven prefectures where the infection had spread [18]. Thereafter, the state of emergency was expanded to all prefectures. However, seven prefectures, including Tokyo, Kanagawa, Saitama, Chiba, Osaka, Hyogo, and Fukuoka, where emergency declarations were issued earlier, had dense populations and were often the subject of intensive emergency declarations. To capture this difference in areas, we grouped the subjects by a risk area, which included the seven mentioned prefectures, and a control area.

The results were expressed by seven coefficients *β*_*0*_-*β*_*7*_ according to this formula.

$$Y_t\:=\:\beta_0\:+\:\beta_1T_t\:+\:\beta_2X_t\:+\:\beta_3X_tT_t\:+\:\beta_4Z\:+\:\beta_5ZT_t\:+\:\beta_6ZX_t\:+\:\beta_7ZX_tT_t\;+\;e_t$$

- β_0_: Estimated the base level;
- β_1_: Estimated the trend pre-COVID-19;
- β_2_: Estimated the change in level post-COVID-19;
- β_3_: Estimated the change in trend post-COVID-19;
- β_4_: Estimated the trend in the risk area;
- β_5_: Estimated the trend pre-COVID-19 in the risk area;
- β_6_: Estimated the change in level post-COVID-19 in the risk area;
- β_7_: Estimated the change in trend post-COVID-19 in the risk area.

Here, Tt is the time elapsed each month (1 ≤ Tt ≤ 24) from the start (December 2018) to the end of the observation period (November 2020). Xt was a dichotomous dummy variable that included pre-COVID-19 (Xt = 0) and post-COVID-19 (Xt = 1). Z was also a dummy variable that denoted the area difference: seven prefectures (Z = 1) and other prefectures (Z = 0). A generalized linear model was applied using log link and Poisson distribution. STATA version 14.2 (Stata-Corp LP, College Station, TX, USA) was used for the analysis. The results of analysis and figures were output via the STATA syntax “itsa” introduced by Linden [19]. The statistical significance level was a two-sided *p*-value of less than 5%.

### Ethical issues

This study was conducted following the guidelines of the Declaration of Helsinki and was approved by the Institutional Review Board (Ethical Review Board in Institution of Medicine, University of Tsukuba. Approval number: 1301). The present study data was completely anonymous without individual information. No personal information was obtained on the part of the researcher for this study. Therefore, disclosure of this study information to the research subjects was done publicly. Patient informed consent was waived by the review board of Institution of Medicine in University of Tsukuba as the data contains no individually identifying information.

## Results

The participating providers were care management providers (*n* = 5,767), home-visit care providers (*n* = 3,506), home-visit nursing providers (*n* = 971), and adult day care providers (*n* = 4,650). The breakdown between the seven risk area prefectures where emergency declarations were issued earlier and the other areas (35 prefectures) was close to 50–50 for all service providers. The risk areas were urban and had a larger number of providers between them. The numbers of providers in the risk areas and control areas are listed in Table 1. For each of the Pre- and Post-COVID-19 periods, we described the mean and median across insurance provider revenues, in 30-day periods. Comparisons of the values between Pre- and Post-COVID-19 showed an increasing trend in all services and areas, except for adult day care in the risk areas. Among adult day care providers in the risk areas, the mean decreased by approximately 100 USD and the median decreased by approximately 200 USD.

**Table 1:** The revenues (USD) of long-term care insurance services pre- and post-COVID-19 in Japan

			Pre-COVID-19 (Nov 2018 - Dec 2019)		Post-COVID-19 (Jan 2020 - Nov 2020)	
			Mean (SD)	Median (IQR)	Mean (SD)	Median (IQR)
Care management	In risk-areas	(*n* = 2,702)	6375.8 (6759.1)	4119.8 (4882.6)	6920.6 (7722.0)	4313.2 (5584.4)
	In other areas	(*n* = 3,065)	5479.9 (5409.0)	3603.1 (4056.1)	5864.9 (5947.1)	3708.2 (4534.0)
Home-visit care	In risk-areas	(*n* = 1,843)	23826.8 (32046.9)	15148.9 (21706.7)	25556.3 (35060.2)	15369.2 (23562.4)
	In other areas	(*n* = 1,663)	20282.8 (24771.3)	12945.9 (19537.3)	21561.4 (26352.7)	13607.6 (20969.0)
Home-visit nursing	In risk-areas	(*n* = 452)	21881.4 (28918.8)	14392.0 (18750.0)	25694.9 (33995.5)	17393.2 (24713.3)
	In other areas	(*n* = 519)	12892.6 (11642.5)	10107.9 (11840.4)	14945.4 (12834.9)	12185.5 (14862.1)
Adult day care	In risk-areas	(*n* = 2,067)	23497.2 (16314.5)	19764.3 (16628.2)	23390.7 (16938.8)	19538.1 (17043.9)
	In other areas	(*n*= 2,583)	23542.6 (16322.4)	19987.5 (18225.6)	23825.6 (16974.5)	20183.5 (19048.3)

*SD* Standard deviation, *IQR* Interquantile range

**Fig. 1:**
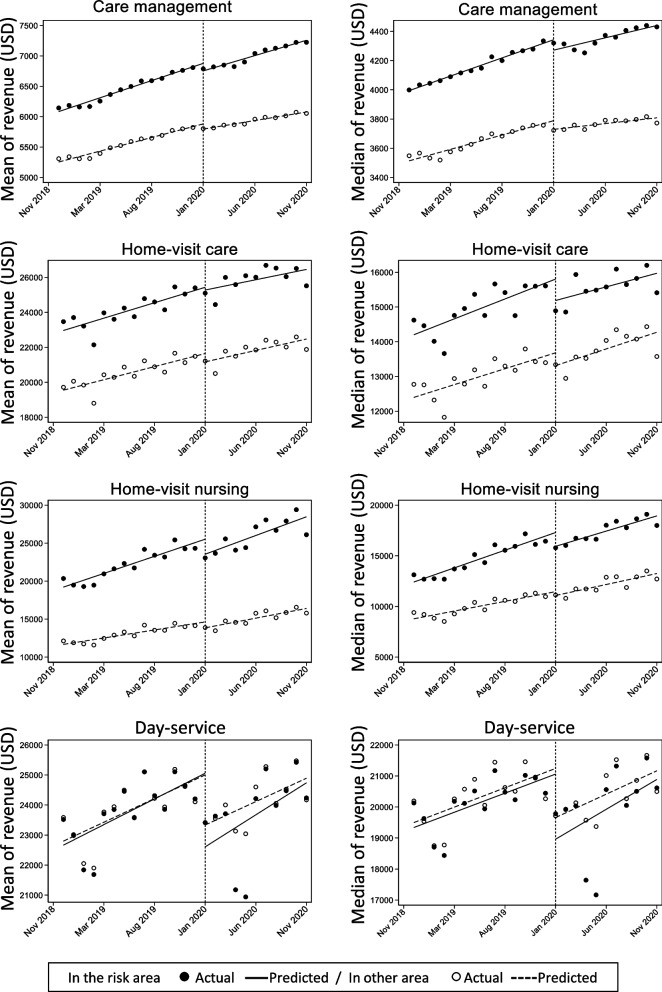
Changes in the revenues (USD) of long-term care insurance services during the COVID-19 pandemic in Japan

In the Fig. 1, the plots show the actual observed values (dots) and the predicted (regression) lines between the risk and control areas. The regression lines expressed an increasing trend in both the means and medians of all the services and areas during all periods. However, in the case of adult day care, the intercept of the line in Post-COVID-19 declined markedly. The lines for the risk areas were also below those of the others. During the Post-COVID-19, the space between lines widened.

Table 2 presents the results of our interrupted time-series analysis. For care management, there was a decline in both the level and trend Post-COVID-19. For home-visit care and home-visit nursing, only a decline in the mean level of home-visit care was observed. For adult day care, there were big drops in the means and medians, but the trends did not change.

**Table 2:** Changes in the revenues (USD) of long-term care insurance services during the COVID-19 pandemic in Japan

	Care management		Home-visit care		Home-visit nursing		Adult day care	
	Mean	Median	Mean	Median	Mean	Median	Mean	Median
Change in level post-COVID-19	-85.1^**^	-59.3^**^	-478.3	-368.3	-779.7^*^	-378.8	-1668.8^**^	-1583.2^**^
Change in trend post-COVID-19	-16.7^**^	-11.7^**^	-18.9	4.3	44.1	25.4	-1.9	26.1
Change in level post-COVID-19 in risk-areas	-38.6	-12.0	322.2	-248.8	-1194.7	-981.7	-782.2	-516.5
Change in trend post-COVID-19 in risk-areas	10.4	3.7	-39.6	-40.4	-2.0	-74.0	44.1	43.5

^**^p < 0.01, ^*^p < 0.05

## Discussion

The results showed that the LTCI revenue of care management providers decreased significantly in terms of both level and trend after the COVID-19 pandemic. The revenue level of home-visit nursing providers also decreased. Finally, in adult day care providers, the level of revenue declined markedly, but the trend did not. In our previous study, the number of users of day services decreased in that trend after the pandemic began. Therefore, as one of the possible reasons, they had restoration of revenue in that trend because the amount of use was increased each individual based on their demands. These nationwide results could be the potential evidence to help the prediction and counter-measurement in the future.

The decrease in the level of revenue among adult day care providers was suggested to have had a serious impact on the sustainability of the business. Prior studies have shown that the decline in the number of adult day care users was significant, which also led to a decrease in revenue for the providers. In adult day care, since people gather and engage in group activities, both users and providers have to be cautious during activities in order to prevent infection. However, the reduction in users may also have influenced the revenue of the providers. Among adult day care providers, approximately 1,600 USD worth of revenue was reduced. A decrease in revenue of about 1,600 USD per month can be equivalent to the salary of one employee [20]. Aramaki [20] mentioned the ratio of labor costs tends to be high among long-term care providers, and in adult day care providers it has been reported to be over 60%. In home-visit nursing, the labor cost ratio is even larger, at over 80%. Therefore, employment adjustments may be necessary even for decreases in revenue at an average level of 779 USD. Reductions in revenue can make it difficult for service providers to maintain operations, as well as retain personnel and staff.

In Japan, several surveys have shown concerns regarding the reduction in revenue of LTCI providers. They reported continued year-over-year declines in expenses for personal preventive equipment (PPE) maintenance [12, 21]. Some reports suggested that the COVID-19 pandemic may have exacerbated the effects of already unstable management practices, resulting in bankruptcies [11, 22]. In the US, the American Health Care Association and the National Center for Assisted Living (AHCA/NCAL) reported that 55% of welfare facilities for older adults operate at a loss, and that 89% of facilities operate at a profit margin of less than 3% [23]. In addition, many facilities have cited increased costs due to the introduction of additional personnel and PPE for infection control [23]. In a survey of home-visit care providers in Massachusetts, 80.9% of the 94 providers who responded reported a decrease in home-visit care hours, 98.7% experienced cancellation of visits due to infection concerns, and 64.5% reported that family members took over caregiving of their patients [24]. The threat of the pandemic forced many lifestyle changes in order to save lives. Among older adults in particular, who are the most at-risk of severe COVID-19 outcomes, changes in the use of long-term care services have been significant. This has affected the management of care centers. Smaller providers in rural areas are more unstable and susceptible to such a decrease in revenue. Typically, these providers are indispensable public resources for maintaining long-term care in the community. Although what we have identified in this study is only a partial decrease in revenue, there is concern that this process will lead to the closure of some providers. It is necessary to continue monitoring the supply of long-term care and the influence of the pandemic. In Japan, financial assistance was provided for LTCI providers suffering from the pandemic, but this was intended to provide necessary PPE and comfort to staff and to supplement insurance, not to compensate for loss of revenue [25]. In the US, the government helps the Medicare service supplier to recoup the claim to support their financial aspects [26]. In Japan, the most important thing was to ensure infection prevention, and the care providers were not able to catch up with changes in their finances. The reduction in revenue was not anticipated during the progression of the pandemic. During a pandemic, such as the one that occurred in this study, a decrease in the revenue of LTCI service providers can be expected. The results of this study can hopefully provide suggestions for countermeasures, in addition to support measures already enacted. The number of users and the revenue decreased due to infection control measures. However, it was necessary to maintain staffing to work for infection control for those who continue to use. For the imbalance between the revenue and the number of people employed, we consider that funds defined in the amount of the decrease in users would ensure the maintenance of conventional employment. Additionally, it would contribute to maintaining the quality of services after the pandemic.

This study had several limitations. First, it was a descriptive study and could not provide causal inferences. The data source was an online support system for LTCI providers in Japan. Although the data were available on a national scale, there are concerns regarding their national representativeness. In particular, the scale of the business of LTCI providers was not considered, and the interpretation of the results should be viewed with caution.

## Conclusion

This study found that after the beginning of COVID-19, there was a marked decline in LTCI revenue at care management, home-visit nursing, and adult day care providers. The downward trend in adult day care was particularly significant, suggesting an impact on the maintenance of operations for these businesses. These nationwide results could be the potential evidence to help the prediction and counter-measurement in the future. Additional research is needed to determine whether these changes in the revenues of LTCI providers will impact the maintenance of their business operations.

## Data Availability

The data that support the findings of this study are available from SMS Co., Ltd. but restrictions apply to the availability of these data, which were used under license for the current study, and so are not publicly available.
